# The identification and characterization of a plant height and grain length related gene *hfr131* in rice

**DOI:** 10.3389/fpls.2023.1152196

**Published:** 2023-03-24

**Authors:** Dengyong Lan, Liming Cao, Mingyu Liu, Fuying Ma, Peiwen Yan, Xinwei Zhang, Jian Hu, Fuan Niu, Shicong He, Jinhao Cui, Xinyu Yuan, Jinshui Yang, Ying Wang, Xiaojin Luo

**Affiliations:** ^1^ State Key Laboratory of Genetic Engineering and Engineering Research Center of Gene Technology (Ministry of Education), School of Life Sciences, Fudan University, Shanghai, China; ^2^ Ministry of Education Key Laboratory for Biodiversity Science and Ecological Engineering, Department of Ecology and Evolutionary Biology, School of Life Sciences, Fudan University, Shanghai, China; ^3^ Institute of Crop Breeding and Cultivation, Shanghai Academy of Agricultural Sciences, Shanghai, China; ^4^ Ministry of Education, Key Laboratory of Crop Physiology, Ecology and Genetic Breeding College of Agronomy, Jiangxi Agricultural University, Nanchang, China

**Keywords:** plant height, grain length, brassinosteroid, auxin, *OsARF17*, *OsBRI1*, rice

## Abstract

Plant height and grain size are important agronomic traits affecting rice yield. Various plant hormones participate in the regulation of plant height and grain size in rice. However, how these hormones cooperate to regulate plant height and grain size is poorly understood. In this study, we identified a brassinosteroid-related gene, *hfr131*, from an introgression line constructed using *Oryza longistaminata*, that caused brassinosteroid insensitivity and reduced plant height and grain length in rice. Further study showed that *hfr131* is a new allele of *OsBRI1* with a single-nucleotide polymorphism (G to A) in the coding region, leading to a T988I conversion at a conserved site of the kinase domain. By combining yeast one-hybrid assays, chromatin immunoprecipitation-quantitative PCR and gene expression quantification, we demonstrated that OsARF17, an auxin response factor, could bind to the promoter region of *HFR131* and positively regulated *HFR131* expression, thereby regulating the plant height and grain length, and influencing brassinosteroid sensitivity. Haplotype analysis showed that the consociation of *OsAFR17^Hap1^*/*HFR131^Hap6^* conferred an increase in grain length. Overall, this study identified *hfr131* as a new allele of *OsBRI1* that regulates plant height and grain length in rice, revealed that brassinosteroid and auxin might coordinate through *OsARF17*–*HFR131* interaction, and provided a potential breeding target for improvement of rice yield.

## Introduction

Rice is among the most important food crops worldwide, providing staple food for more than half of the world’s population (Qiao et al., 2021; Huang et al., 2022). However, the global population is growing rapidly and is forecast to attain 9.7 billion by 2050 and 10.4 billion by 2100 (Economic and Social Affairs, 2022). Furthermore, with ongoing economic development, the environment is deteriorating, and the arable land area is decreasing. Therefore, it is particularly important to increase the rice yield per unit land area to keep pace with the projected population growth (McClung, 2014).

Plant height and grain size are two important factors affecting rice yield. An excessively high or low plant height will affect rice yield. A plant that is too tall has poor lodging resistance, whereas if the plant is too short, the plant will produce smaller grains, an increased number of ineffective tillers, and have poor resistance to disease (Liu et al., 2018). Therefore, an appropriate plant height is essential to improve rice yield. This is comparable to the “Green Revolution” wave of semi-dwarf breeding in the 1960s, which doubled food production in much of the world (Hargrove and Cabanilla, 1979; Khush, 1999). The yield per plant of rice is directly affected by three factors: panicle number, number of grains per panicle, and thousand-grain weight. The thousand-grain weight is positively associated with grain size, including grain length, grain width, grain thickness, and degree of grain filling (Xing and Zhang, 2010). Thus, grain size is among the most agronomically important traits in rice breeding.

Plant hormones play crucial roles in the regulation of plant height and grain size in rice, among which brassinosteroids (BRs) are particularly important. Similar in structure to mammalian steroid hormones, BRs are a class of polyhydroxysteroid plant hormones and are involved in multiple biological processes in growth and development (Clouse, 2011). Many BR-related genes have been reported in rice, and most affect both plant height and grain size. The BR synthesis-associated mutants *brd2*, *d11*, *d2*, and *brd1* have a reduced plant height and grain size to different degrees (Mori et al., 2002; Hong et al., 2003; Hong et al., 2005; Tanabe et al., 2005). *OsBRI1*, the rice ortholog of *Arabidopsis BRI1*, encodes the receptor of BR in rice. Loss-of-function of *OsBRI1* results in dwarf culms, reduced grain size, erect leaves, and insensitivity to BR (Yamamuro et al., 2000; Morinaka et al., 2006). OsBAK1 interacts with OsBRI1 to promote BR signal transduction downstream. Overexpression of *OsBAK1* increases sensitivity to BR, however, reduces plant height and grain size, which may be caused by repressing GA level (Li et al., 2009; Tong et al., 2014). O*sGSK1*–*OsGSK4*, which are homologs of *AtBIN2*, belong to the GSK3-like kinase family and play an important role in the regulation of BR signaling (Yoo et al., 2006). Loss-of-function of one or several *OsGSK* genes has revealed that the *gsk2*, *gsk1,2*, *gsk2,3*, and *gsk2,4* mutants show increased plant height, whereas the *gsk2,3,4* mutant has decreased plant height and that of the *gsk1,2,3,4* mutant is not changed significantly. However, the grain length and thousand-grain weight of all types of mutants is significantly increased (Liu et al., 2021). The mutant or knockout lines of the BR-associated transcription factors *OsBZR1*, *OsLIC*, and *DLT*/*GS6* all show significant dwarfing; however, the grain size and yield of the mutant or knockout lines of *OsBZR1* and *OsLIC* are significantly decreased, whereas those of the *gs6* mutant are significantly increased (Bai et al., 2007; Wang et al., 2008; Tong et al., 2009; Sun et al., 2013; Zhu et al., 2015). Other BR regulators, such as *OsBU1* and *SG1*, are also involved in the regulation of plant height and grain shape (Tanaka et al., 2009; Nakagawa et al., 2011). BR plays an important role in the regulation of multiple phenotypes in rice, such as plant height, grain size, seed germination, etc, involved in complex molecular regulatory networks (Xiao et al., 2020; Niu et al., 2022; Xiong et al., 2022), which is worth of further study. In addition, multiple hormones regulate the plant height and grain size of rice, but how BR coordinates with other hormones requires further study.

In this study, we identified a gene, *HFR131*, that regulates plant height and grain length in rice. Further study of the *hfr131* mutant demonstrated that *hfr131* is a new allele of *OsBRI1*. The replacement of amino acids in the kinase domain of HFR131 caused dwarfing and reduced grain length in the near-isogenic line NIL-*hfr131*. The auxin response factor OsARF17 was found to regulate plant height and grain length through BR signaling by binding to the promoter of *HFR131* and thereby regulating its expression. In addition, the consociation of *OsAFR17^Hap1^*/*HFR131^Hap6^* was confirmed to increase the grain length.

## Materials and methods

### Plant materials and growth conditions

**Figure 1:**
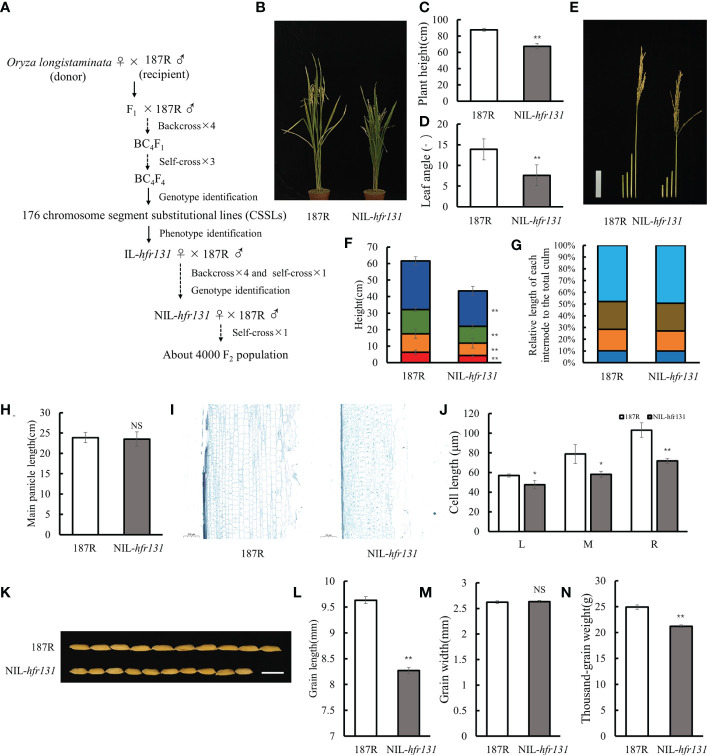
Phenotypes of rice ‘187R’ and NIL-*hfr131*. **(A)** Construction of infiltrating lines and F_2_ population. **(B–D)** Plant architecture, plant height, and the third leaf angle of 187R and NIL-*hfr131*. **(E–G)** Internode morphology (bar = 10 cm), internode length, and internode elongation pattern of 187R and NIL-*hfr131*. **(H)** Panicle length of 187R and NIL-*hfr131*. **(I, J)** Longitudinal sections of the stem and internode cell length (L, M, and R refer to the left, middle, and right side of the section, respectively) of 187R and NIL-*hfr131* (bar = 200 μm). **(K–N)** Grain morphology (bar = 1 cm), grain length, grain width, and thousand-grain weight of 187R and NIL-*hfr131*. * *P* < 0.05, ** *P* < 0.01, NS non-significant.

Using wild rice *Oryza longistaminata* as the donor parent (♀) and *indica* restorer rice ‘187R’, cultivated by Guangxi Academy of Agricultural Sciences, as the recipient parent (♂), the BC_4_F_1_ population was obtained through hybridization and backcrossing with 187R (♂) for four successive generations and the BC_4_F_4_ population was obtained by selfing the BC_4_F_1_ population for three successive generations. Then we identified the genotype of BC_4_F_4_ population with genetic markers and a set of introgression lines were constructed (**Figure 1A**). Among these lines, by phenotype identification, we identified an introgression line, IL-*hfr131*, that exhibited significantly reduced plant height, small grain size and erect leaf. The alleles in IL-*hfr131* and 187R were therefore designated *hfr131* and *HFR131*, respectively. Subsequently, we backcrossed IL-*hfr131* (♀) with its recurrent parent 187R (♂) for four successive generations and selfed for one generation, then identified the genotype of the offspring with genetic markers, obtaining a near-isogenic line, NIL-*hfr131*, with an introgression fragment on the long arm of chromosome 1 (**Supplementary Figure 1**). Next, NIL-*hfr131* was used to construct a F_2_ population comprising approximately 4000 plants for fine-mapping and phenotypic analysis (**Figure 1A**). The plant height (*n* = 13), leaf angle (*n* = 10), internode length (*n* = 13) and panicle length (*n* = 13) of 187R and NIL-*hfr131* were measured. For grain length, grain width and grain weight, each variable was measured on three groups with 50 seeds per group.

All rice materials in this study were planted in Shanghai (31°20′26″ N, 121°30′26″ E) in summer and in Hainan (18°18′52″ N, 109°03′05″ E) in winter. Standard field management practices were applied for all lines.

### Histological analysis

The second internode of rice stems was sampled and fixed with 50% FAA solution (50 mL anhydrous ethanol, 10 mL 37% formaldehyde, 5 mL glacial acetic acid, 35 mL ddH_2_O). Histological sections of the samples were prepared by the Wuhan Misp Biotechnology Co., Ltd. (Wuhan, China). The internode cell length of the left, middle, and right side of the section were measured and three layers of cells were measured in each region.

### BR treatment

Rice seeds were cultured in 0.5% agar semi-solid medium containing 0 or 1 μM/L 24-epibrassinolide (24-eBL) (Zhang et al., 2015), which is commonly used to test the responsiveness of plants to BR, for 1 week in a greenhouse at 26 °C under shading. The coleoptile length and root length were then measured (*n* = 10). The change in root length was calculated as a measure of the degree of inhibition of root elongation (*n* = 10) using the following formula: (root length with 1 μM/L 24-eBL treatment − root length without 24-eBL treatment)/root length without 24-eBL treatment.

### Map-based cloning

Total DNA was extracted from leaf samples. The corresponding markers were amplified by PCR, and the PCR products were separated by polyacrylamide gel vertical electrophoresis. The primers used for fine mapping are listed in **Supplementary Table 3**.

### RNA isolation and quantitative real-time PCR

Total RNA from rice samples was extracted using the FastPure^®^ Plant Total RNA Isolation Kit (Vazyme, Nanjing, China). First-strand cDNA was generated by reverse transcription using the PrimeScript™ RT Reagent Kit (Takara, Otsu, Japan). Quantitative real-time PCR (qPCR) was conducted using TB Green Premix Ex Taq II (Tli RNaseH Plus) (Takara) and a CFX96 Real-Time PCR Detection system (Bio-Rad, Hercules, CA, USA). The reactions were conducted with 40 cycles of 95°C for 5 s and 60°C for 30 s. Melting curves were prepared and analyzed. *OsActin* or *OsUBQ5* was used as an internal reference gene. Three biological replicates were performed for each sample. The primers used are listed in **Supplementary Table 3**.

### Dual-luciferase reporter assay

The promoter fragment of *HFR131* in the same region of 187R (3500 bp) and NIL-*hfr131* (3067 bp) was cloned and inserted into the *pGreenII 0800-LUC* vector using seamless cloning technology to construct the recombinant vectors *proHFR131-LUC* and *prohfr131-LUC*. Seeds of rice ‘9311’ were germinated on wet napkins at 37 °C for 14 days. The stem of the seedlings was cut into thin slices and protoplasts were prepared by hydrolyzing the cell walls with cellulase and macerozyme. The recombinant vectors were transferred into the ‘9311’ protoplasts using 40% polyethylene glycol solution and cultured at 28 °C for 12–16 h. The protoplast lysate was collected and the promoter activity of the *HFR131* promoter was tested using the Dual-Luciferase^®^ Reporter Assay System (Promega, Madison, WI, USA) and a Synergy™ 2 Multi-Mode Reader (BioTek, Winooski, VT, USA). The activation efficiency of luciferase was expressed as the ratio of firefly luciferase (LUC) to *Renilla* luciferase (REN); the higher the ratio, the stronger the promoter activity. Three technical replicates were performed for each sample. The primers used are listed in **Supplementary Table 3**.

### Transcriptome analysis

Total RNA was extracted from young panicles (*n* = 3) before heading date of 187R and NIL-*hfr131* using the FastPure^®^ Plant Total RNA Isolation Kit (Vazyme). The extracted RNA was used as the template for construction of cDNA libraries, which were sequenced using a MGI MGISEQ-2000 platform by Sangon Biotech (Shanghai, China). The relative gene expression level was calculated and normalized to fragments per kilobase of transcript per million mapped reads. Differentially expressed genes (DEGs) were annotated and classified according to their putative or proven function. The transcriptome datasets were submitted to the GEO database as accession nos. GSE224118.

### Construction of transformation lines

Overexpression lines of *HFR131* were based on the NIL-*hfr131* mutant background. For the coding sequences (CDSs) of *OsARF17* and the promoter sequences of *HFR131* in ‘ZH11’ and 187R were consistent, respectively, and ZH11 had high seed setting rate, which helped us investigate the phenotype of the grains well, we construct *OsARF17* knockout and overexpression lines based on ZH11 background. CRISPR/Cas9 knockout lines of *OsARF17* were generated by BIOGLE GeneTech (Jiangsu, China), and the target sequence was 5′- CCAATTATCCTAACTTGCCTCCACAACTTATTTGCCAACTTCACAATGTGACG-3′. All knockout lines of the target sequence were cloned by PCR and sequenced. Regarding the overexpression lines, the CDSs of *OsARF17* (LOC_Os06g46410.1) and *HFR131* (LOC_Os01g52050.1) were cloned and inserted into the *PCHF-FLAG* vector to construct the overexpression vectors *CaMV35-OsARF17-FLAG* and *CaMV35S-HFR131-FLAG* using a seamless cloning technique. *CaMV35S-OsARF17-FLAG* was transformed by BIOGLE GeneTech and *CaMV35S-HFR131-FLAG* was transformed by BioRun (Wuhan, China). All transformed lines were verified by qPCR, and eight biological replicates were performed for *OsARF17* overexpression lines (T_2_ generation) and three technical replicates were performed for *HFR131* overexpression lines (T_0_ generation). The primers used are listed in **Supplementary Table 3**. The plant height, grain length and grain width of transformation lines were measured. Ten biological replicates were performed for T_3_ generation of *OsARF17* knockout lines and T_2_ generation of *OsARF17* overexpression lines. For the grain length and thousand-grain weight of T_0_ generation of *HFR131* overexpression lines, each variable was measured on three groups with 20 seeds per group.

### Yeast one-hybrid assay

Eight 150–300 bp DNA fragments containing an auxin response element (AuxRE) in the 3500 bp promoter region of *HFR131* were cloned and inserted into the *pAbAi* vector. The recombinant vectors were transferred into the yeast strain Y1H Gold, and cultured and screened on SD−Ura solid medium. The positive clones were detected by colony PCR using the primers Y1-test and pAbAi (**Supplementary Table 3**). The positive colonies were diluted and cultured in SD−Ura solid medium containing aureobasidin A (ABA^r^) at different concentrations ranging from 0 to 1000 ng/ml. The culture with the optimal concentration of ABA^r^ was selected to eliminate self-activation. The CDS of *OsARF17* was cloned by PCR and inserted into the *pGADT7* vector to construct the *AD-ARF17* recombinant vector, which was transformed into yeast Y1H Gold containing one of the DNA fragments mentioned above. The transformed Y1H Gold cells were cultured on SD−Leu solid medium containing the selected ABA^r^ concentration. The empty *pGADT7* vector, *KAD*, was transformed as a negative control. The Yeastmaker Yeast Transformation System 2 (Clontech) was used following the manufacturer’s instructions. The primers used are listed in **Supplementary Table 3**.

### ChIP-qPCR

Approximately 2.5 g rice seeds were weighed, soaked in water at 37 °C for 24 h, and then sown in nutrient soil 48 h after germination. The seeds were incubated at 26 °C for 14 days and then used for chromatin immunoprecipitation–quantitative real-time PCR (ChIP-qPCR). The experimental procedure followed the method of Wang et al. (Wang et al., 2018). The antibody used in this study was the FLAG antibody and the magnetic beads used were protein A/G magnetic beads. *OsActin* was used as an internal standard for data normalization. Three technical replicates were performed for each sample. The primers used are listed in **Supplementary Table 3**.

### Haplotype analysis

A haplotype network for *HFR131* and *OsARF17* based on pairwise differences between two adjacent gene–CDS–haplotypes (gcHaps) was constructed using the pegas R package. The gcHaps data used for building the network were downloaded from the Rice Functional Genomics and Breeding (RFGB) database (https://www.rmbreeding.cn). The 3K Phenotype data were downloaded from RFGB (https://www.rmbreeding.cn/Phenotype). One-way analysis of variance (ANOVA) of the gcHap data was conducted for three agronomic traits (plant height, grain length, and thousand-grain weight), followed by Duncan’s multiple-range test for multiple comparisons using the R package agricolae (v.1.3-5). Two-way ANOVA of the gcHaps data was conducted for analysis of grain length, followed by Duncan’s multiple-range test for multiple comparisons using the R package agricolae (v.1.3-5). The probability level *P* < 0.05 was considered to be statistically significant. The multiple comparisons and the distribution of gcHaps among the five major populations, *Aus*, *Bas*, *XI*, *GJ* and *Adm*, were visualized using the R package ggplot2 (v.3.4.0) as boxplots and stacked barplots. The single-nucleotide polymorphism (SNP) dataset used for calculation of nucleotide diversity (π) and Tajima’s *D* was downloaded from the Rice SNP-Seek Database (https://snp-seek.irri.org/_download.zul). Nucleotide diversity (π) at each window in the target region was estimated using VCFtools (v.0.1.15) with the “–window-pi” and “–window-pi-step” options, and the average value was used as its *p*-value. Tajima’s *D* for the target region was calculated using VCFtools (v.0.1.15) with the “–TajimaD” option.

## Results

### The near-isogenic line NIL-*hfr131* exhibited reduced plant height and grain length

Compared with cultivated rice, wild rice has a larger gene pool, which allows adaptation to different environments and is a valuable resource for gene functional research in rice. In this study, we identified an introgression line, IL-*hfr131*, with significantly reduced plant height and grain size using wild rice *Oryza longistaminata* as the donor parent (♀) and *indica* restorer rice 187R as the recipient parent (♂) (**Figure 1A**), and further constructed a near-isogenic line, NIL-*hfr131*, containing the gene *hfr131* (**Supplementary Figure 1**). Subsequently, a F_2_ population containing approximately 4000 plants was constructed using NIL-*hfr131* (♀) and 187R (♂) (**Figure 1A**).

Phenotypic investigation showed that the ratio of dwarf and small-grained plants to high and big-grained plants in the F_2_ population was close to 1:3, indicating that *hfr131* was a recessive gene. Compared with 187R, NIL-*hfr131* showed a dwarf plant height accompanied by erect leaves (**Figures 1B–D**). To determine the direct cause of the plant height reduction, we measured the length of the first four internodes (counting the internode directly connected to the panicle as the first internode) and the panicle length of 187R and NIL-*hfr131*. The results showed that each internode of NIL-*hfr131* was significantly shortened to equal proportions (**Figures 1E–G**). However, there was no significant difference in panicle length between 187R and NIL-*hfr131* (**Figure 1H**), indicating that the reduced plant height of NIL-*hfr131* was caused by the equally proportional shortening of the internodes. The length of the internode is directly related to the growth and development of the internode cells. To further clarify the mechanism of internode shortening of NIL-*hfr131*, longitudinal sections of the internodes of NIL-*hfr131* and 187R were prepared. The results showed that the stem cell length of NIL-*hfr131* was significantly shortened both externally and internally (**Figures 1I, J**), indicating that the reduced internode length of NIL-*hfr131* was mainly caused by shortening of the cell length in the stem. Although the panicle length of NIL-*hfr131* was not changed significantly, the grain size was significantly decreased. Compared with 187R, the grain length and thousand-grain weight of NIL-*hfr131* were significantly decreased, but there was no significant difference in grain width (**Figures 1K–N**). Taken together, these results showed that the reduced plant height and grain size of NIL-*hfr131* was caused by shortening of the internodes and grain length, respectively.

### NIL-*hfr131* is insensitive to brassinosteroid

**Figure 2:**
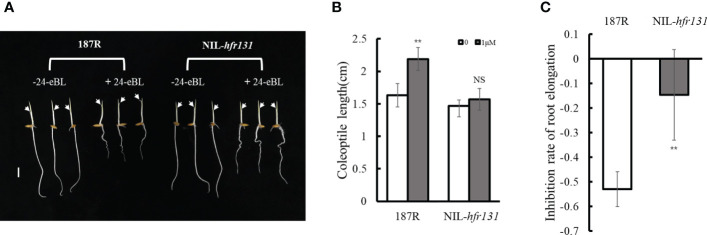
Responses of rice ‘187R’ and NIL-*hfr131* to 24-epibrassinolide (24-eBL). **(A)** Effect of 24-eBL on coleoptile and root elongation in seedlings. The arrows indicate the top of the coleoptile. Bar = 1 cm. **(B)** Coleoptile length of 187R and NIL-*hfr131* seedlings in the presence or absence of 1 μM/L 24-eBL. **(C)** Inhibition of root elongation of 187R and NIL-*hfr131* in response to 1 μM/L 24-eBL. ** *P* < 0.01, NS non-significant.

Given that NIL-*hfr131* plants were reduced in height and had erect leaves, which is similar to the mutant phenotype of genes associated with the BR synthesis and signaling pathways (Hong et al., 2005; Nakamura et al., 2006; Tong et al., 2009), we speculated that *HFR131* may be involved in the BR synthesis or signaling pathways. To test this hypothesis, we conducted a BR treatment assay. The results showed that the coleoptile length of 187R was significantly increased after 24-eBL treatment, whereas NIL-*hfr131* showed no significant change (**Figures 2A, B**). Furthermore, after 24-eBL treatment, the root length of 187R was extremely reduced (**Figure 2A**). Although the root of NIL-*hfr131* also showed significant shortening, the inhibitory effect of 24-eBL on NIL-*hfr131* was weaker than on 187R (**Figures 2A–C**). In conclusion, NIL-*hfr131* was insensitive to BR, suggesting that *HFR131* was involved in the BR signaling pathway rather than the BR synthesis pathway.

### *hfr131* is a new allele of *OsBRI1*

**Figure 3:**
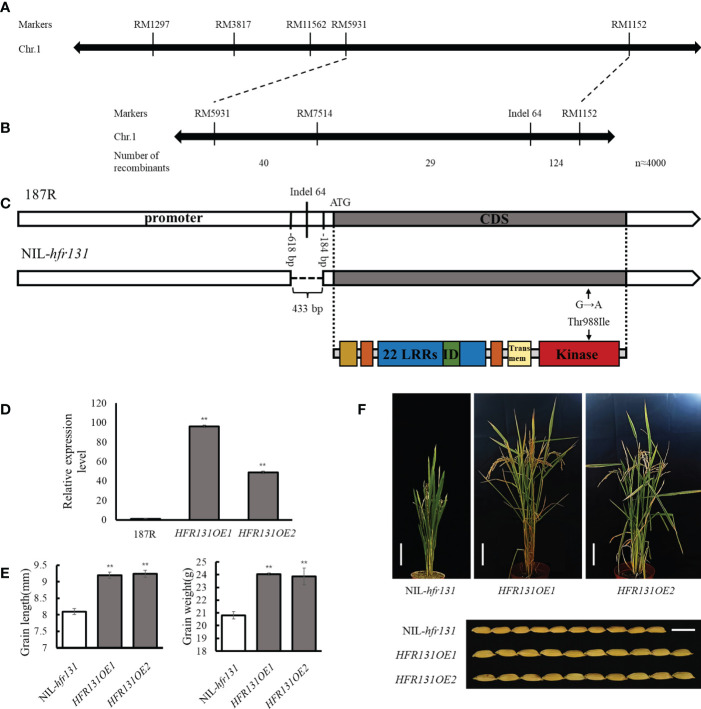
Identification of *HFR131*. **(A)** Linkage map of *HFR131*. **(B)** Fine mapping of *HFR131* using almost 4000 F_2_ plants. The number of recombinants between adjacent markers is indicated under the linkage map. **(C)** Gene structure of *OsBRI1* in rice ‘187R’ and NIL-*hfr131*, and the corresponding protein structure redrawn from Nakamura et al. (Nakamura et al., 2006). Positions of the 433 bp deletion are presented by base number before the ATG. **(D)** Transgene expression level of *OsBRI1*/*HFR131*. *OsActin* was used as an internal reference. **(E)** Grain length and thousand-grain weight of NIL-*hfr131* and *HFR131* overexpression lines (T_0_ generation). **(F)** Plant architecture (Bar = 10 cm) and grain morphology (Bar = 1 cm) of NIL-*hfr131* and *HFR131* overexpression lines (T_0_ generation). The plant height of NIL-*hfr131*, *HFR131OE1* and *HFR131OE2* is 50.9 cm, 68 cm and 66 cm, respectively. ** *P* < 0.01.

To map the location of the *HFR131* locus, we constructed two pools comprising 36 dwarf small-grained plants (DS; recessive plants) and 36 tall large-grained plants (HB; dominant plants), selected from the F_2_ population, and analyzed the two pools using simple sequence repeat (SSR) markers distributed on chromosome 1. The markers RM1297 and RM5931 showed polymorphism between the pools, and exhibited markedly different banding patterns between recessive individuals and dominant individuals (**Supplementary Figure 2**), indicating the two markers were closely linked to *HFR131*. Combining these two markers and neighboring SSR markers for further analysis, we preliminarily narrowed the effective locus to the interval between markers RM5931 and RM1152 (**Figure 3A**).

To fine map *HFR131*, we used RM5931 and RM1152 to screen 926 recessive plants selected from the F_2_ population of almost 4000 plants, and 193 recombinants were identified. The SSR marker RM7514, located between RM5931 and RM1152, was used to analyze the 193 recombinants. The *HFR131* locus was located in the region between RM7514 and RM1152. In addition, Indel 64 was designed based on sequence differences between 187R and NIL-*hfr131*, and was found to co-segregate with NIL-*hfr131* (**Figure 3B**), indicating that *HFR131* was located at or adjacent to the site of Indel 64. From a BLAST search of the National Center for Biotechnology Information databases (https://www.ncbi.nlm.nih.gov/), we found that Indel 64 was located in the promoter region of *OsBRI1*, implying that *OsBRI1* may be the candidate gene for *HFR131*.

We then sequenced the promoter and coding regions of *OsBRI1* of 187R and NIL-*hfr131*. Two main mutant sites were found. One mutation was a 433 bp deletion in the promoter region, and the other was a SNP (G to A transition) in the coding region, converting threonine to isoleucine at position 988 in the kinase domain of OsBRI1 (**Figure 3C**). These results indicated that *HFR131* may be *OsBRI1*.

To investigate whether *OsBRI1* correspond to the phenotypic change in NIL-*hfr131*, we overexpressed the wild-type *OsBRI1*, cloned from 187R, in NIL-*hfr131* (**Figure 3D**). The results showed that, compared with NIL-*hfr131*, the plant height, leaf angle, grain length, and thousand-grain weight of the overexpression lines (*HFR131OE1* and *HFR131OE2*) were significantly increased (**Figures 3E, F**), indicating that *HFR131* is *OsBRI1* and that *hfr131* is a new allele of *OsBRI1*.

### Amino acid non-synonymous substitution in the HFR131 kinase domain resulted in the reduction of plant height and grain length

**Figure 4:**
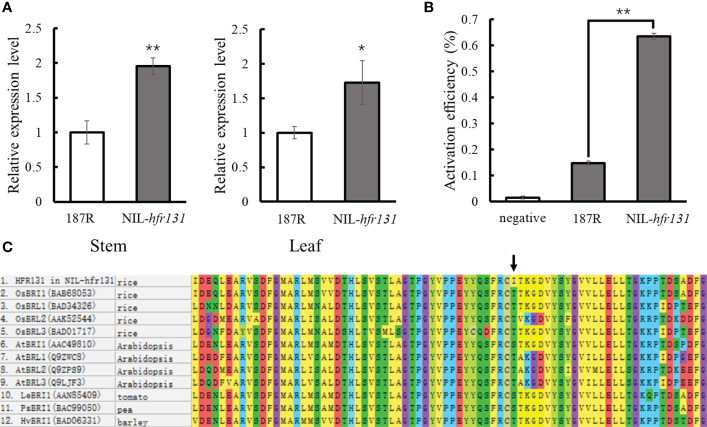
Analysis of the effective mutation site. **(A)** Expression level of *HFR131* in the stem and leaf of 187R and NIL-*hfr131*. **(B)** Detection of promoter activity. Activation efficiency was calculated as LUC/REN. **(C)** Alignment of a portion of the kinase domain of *Arabidopsis thaliana*, rice, tomato, pea, and barley BRI family members. The arrow indicates the mutation site. * *P* < 0.05, ** *P* < 0.01.

Given that there were two mutation sites in *hfr131*, to determine which of the sites (the SNP in the coding region or the 433 bp deletion in the promoter region) was responsible for the height reduction and short grain length of NIL-*hfr131*, we first detected the expression levels of *HFR131* in the stem and leaf of 187R and NIL-*hfr131* by qPCR. Deletion of large fragments in the promoter region often leads to a significant reduction in the gene transcript level, resulting in abnormal gene function. Interestingly, compared with 187R, expression levels of *HFR131* in the stem and leaf of NIL-*hfr131* were not decreased, but in both were up-regulated significantly (**Figure 4A**). To further verify whether the 433 bp deletion in the *HFR131* promoter enhanced the promoter activity, we conducted a dual-luciferase reporter assay. We constructed two recombinant vectors, *proHFR131-LUC* and *prohfr131-LUC*, and transferred them into rice protoplasts. The empty vector was transferred as a negative control. The results showed that the luciferase activation efficiency of the *prohfr131-LUC* construct was significantly higher than that of the *proHFR131-LUC* construct (**Figure 4B**). This result suggested that the 433 bp deletion in the *HFR131* promoter region did enhance the promoter activity.

To determine which mutation site was the direct factor causing the phenotypic effects of NIL-*hfr131*, we considered the following three points. First, *hfr131* is a recessive gene. Generally, an increase in the expression of a recessive gene does not cause phenotypic effects. Second, we compared the amino acid sequence of HFR131 in NIL-*hfr131* with homologous proteins of *Arabidopsis*, rice, tomato, pea, and barley, and found that the threonine at position 988 of HFR131 is highly conserved (**Figure 4C**). The mutation site is located in the kinase domain of HFR131, a domain critical to BR signal transduction. However, the amino acid at position 988 of HFR131 was changed to isoleucine in NIL-*hfr131*, a residue with a quite different structure and physical properties to threonine, implying that this site was the target site causing the phenotypic change of NIL-*hfr131*. Third, the overexpression of *HFR131* in NIL-*hfr131* resulted in a significant increase in plant height and grain length (**Figures 3E, F**), indicating that the increased expression level caused by the deletion of 433 dp in the promoter region of *HFR131* was not the direct cause of the decrease in plant height and grain length of NIL-*hfr131*. In conclusion, the amino acid non-synonymous substitution in the kinase domain of HFR131 was the effective site that led to the decrease in plant height and grain length.

To determine how *hfr131* affects plant height and grain length, we selected young panicles before heading date of NIL-*hfr131* and 187R for transcriptome analysis and 2444 differentially expressed genes (DEGs) were detected. Among the DEGs, we found that the expression levels of *D2*, *D11*, and *BRD1*, which are associated with BR synthesis, were significantly increased (**Supplementary Table 1**), which was consistent with the hypothesis of Yamamuro et al. (2000) that plants could compensate for the defects caused by decreased BR sensitivity by increasing BR synthesis. We also observed that genes associated with plant height and grain size showed a significant change in expression level. The positive regulators of grain length *GS2* (Hu et al., 2015) and *OsLG3* (Yu et al., 2017) were significantly down-regulated. The negative regulators of plant height and grain length *OML4* (Lyu et al., 2020) and *OsREM4.1* (Gui et al., 2016) were significantly up-regulated. The expression level of *OsAP2-39* (Yaish et al., 2010) and *OsCYP96B4* (Ramamoorthy et al., 2011), elevating whose expression level would result in dwarf plant height, was significantly upregulated, whereas the expression of *LHD2* (Xiong et al., 2006), a positive regulator of plant height, was significantly decreased (**Supplementary Table 1**). These genes may be the response genes downstream of *HFR131* involved in regulating plant height and grain length.

In addition, hormone-related genes were differentially expressed; for example, the gibberellin (GA)-related gene *OsGA2ox10* (Lo et al., 2008) was significantly up-regulated and the auxin-related genes *OsPIN1d*, *OsPIN8*, and *OsAUX2* (Wang et al., 2009; Yu et al., 2015) were significantly down-regulated (**Supplementary Table 1**), indicating that *HFR131* regulated plant height and grain length by coordinating with other hormones.

### *OsARF17* is involved in the regulation of plant height and grain length by the BR signaling pathway through regulating the expression of *HFR131*

*OsARF17* plays an important role in the regulation of rice architecture, such as leaf angle and tiller angle (Chen et al., 2018; Li et al., 2020; Huang et al., 2021), suggesting that *OsARF17* may be an important target gene in rice breeding. However, whether *OsARF17* is also involved in the regulation of plant height and grain size, two important agronomic traits, has not been reported. It has been reported that *OsARF11* and *OsARF19* can regulate the expression of *OsBRI1* (Sakamoto et al., 2013; Zhang et al., 2015), so we speculated that *OsARF17* may regulate plant height and grain size through *HFR131*.

**Figure 5:**
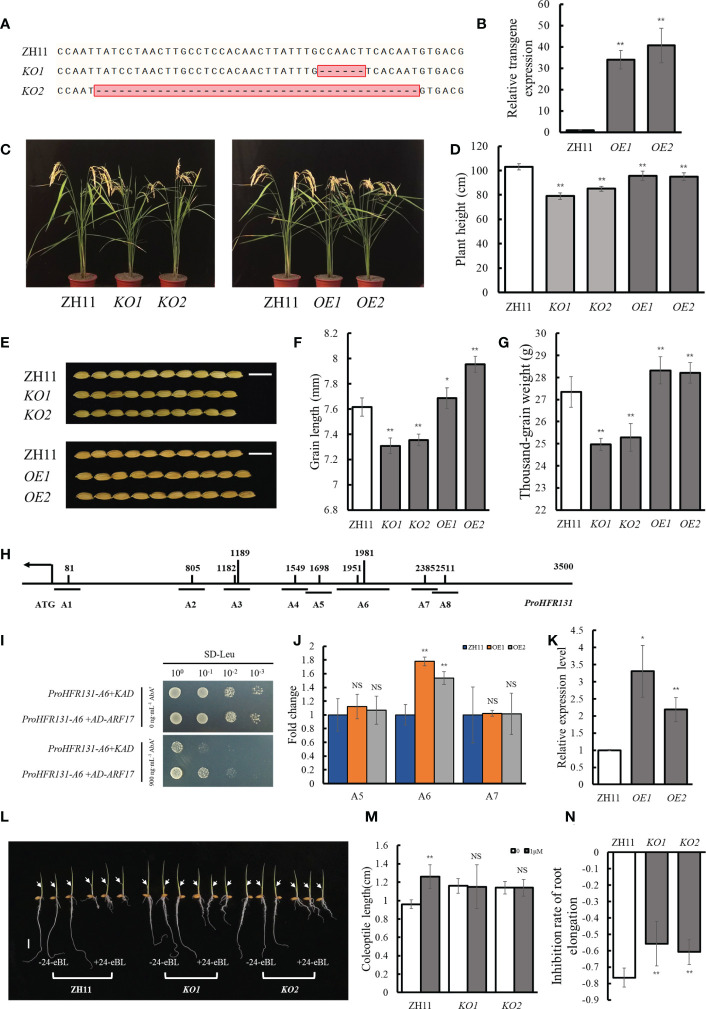
Relationship between *OsARF17* and *HFR131*. **(A)** Target site in the *OsARF17* sequence of rice ‘ZH11’ in the *OsARF17* knockout lines. **(B)** Transgene expression level of *OsARF17*. *OsActin* was used as an internal reference. **(C, D)** Plant architecture and plant height of *OsARF17* knockout lines and overexpression lines. **(E)** Grain morphology of *OsARF17* knockout lines and overexpression lines. Bar = 1 cm. **(F, G)** Grain length and thousand-grain weight of *OsARF17* knockout lines and overexpression lines. **(H)** Positions of 10 auxin response elements in the *HFR131* promoter. **(I)** Yeast one-hybrid assays to detect interaction between OsARF17 and the promoter of *HFR131*. **(J)** ChIP-qPCR analysis to detect interaction between OsARF17 and the promoter of *HFR131*. Fold change is relative to ZH11. *OsActin* was used as an internal reference. A5, A6, and A7 corresponding to the fragments indicated in **Figure 5A**. **(K)** Relative expression level of *HFR131* in *OsARF17* overexpression lines. *OsUBQ5* was used as an internal reference. **(L)** Effect of 24-eBL on coleoptile and root elongation in seedlings. The arrows indicate the top of the coleoptile. Bar = 1 cm. **(M)** Coleoptile length of ZH11 and *OsARF17* knockout-line seedlings in the presence or absence of 1 μM/L 24- epibrassinolide (24-eBL). **(N)** Inhibition of root elongation in ZH11 and *OsARF17* knockout lines under 1 μM/L 24-eBL treatment. * *P* < 0.05, ** *P* < 0.01, NS non-significant.

We constructed two *OsARF17* knockout (*KO1* and *KO2*) and overexpression lines (*OE1* and *OE2*), respectively (**Figures 5A, B**). Phenotypic observation showed that *OsARF17* knockout lines were significantly reduced in plant height compared with ZH11, but *OsARF17* overexpression lines did not show the opposite phenotype (**Figures 5C, D**), which may be related to the interaction of OsARF17 with other proteins in the ARF family, such as OsARF19, whose overexpression lines showed a reduced plant height (Zhang et al., 2015). Whatever, this result showed that *OsARF17* was necessary for the maintenance of plant height in rice. Compared with ZH11, the *OsARF17* knockout lines had significantly shorter grains and decreased thousand-grain weight, whereas *OsARF17* overexpression lines had significantly longer grains and increased thousand-grain weight (**Figures 5E–G**). These results suggested that *OsARF17* affected both plant height and grain length, which was similar to *HFR131*, indicating that *OsARF17* may regulate plant height and grain length through *HFR131*.

To verify whether *OsARF17* can regulate *HFR131*, we searched for TGTCT(A/C)C auxin response elements (AuxREs), which are bound by auxin response factors (ARFs) and confer auxin responsiveness (Wang et al., 2007; Shen et al., 2010), within 3500 bp region of *HFR131* promoter and 10 AuxREs were found (**Figure 5H**). We divided the 10 AuxREs into eight fragments with lengths of 150–300 bp (A1–A8) (**Figure 5H**) and verified by yeast one-hybrid assay and ChIP-qPCR. The yeast one-hybrid assay showed that yeast co-transformed with *ProHFR131-A6* and *AD-ARF17* grew strongly in the selective medium, whereas yeast co-transformed with *ProHFR131-A6* and *KAD* grew weakly (**Figure 5I**). The ChIP-qPCR analysis showed that the A6 fragment of the *HFR131* promoter was significantly enriched in the two overexpressed lines (**Figure 5J**). These results indicated that OsARF17 could bind to the A6 fragment of the *HFR131* promoter. A qPCR analysis revealed that the expression level of *HFR131* was significantly increased in *OsARF17* overexpression lines compared with that of the control ZH11 (**Figure 5K**). These results showed that OsARF17 could bind to the promoter of *HFR131* and positively regulated the expression of *HFR131*.

To further verify whether *OsARF17* was involved in the regulation of *HFR131*, we conducted a BR treatment assay. The results showed that, after treatment with 1 μM/L 24-eBL, root elongation was inhibited more slightly in *OsARF17* knockout lines than in the control ZH11. The length of the coleoptile of ZH11 was significantly increased, whereas no significant change was observed in the *OsARF17* knockout lines (**Figures 5L–N**), indicating that plant sensitivity to BR was decreased after *OsARF17* knockout. These results indicated that *OsARF17* was involved in BR response.

Based on these results, we concluded that OsARF17 could regulate plant height and grain length through regulating the expression of *HFR131*, that *OsARF17* may participate in BR response through *HFR131*, and that *OsARF17*–*HFR131* interaction provides a bridge for communication between auxin and BR.

### Evolutionary correlation analysis of *HFR131* and *OsARF17*

**Figure 6:**
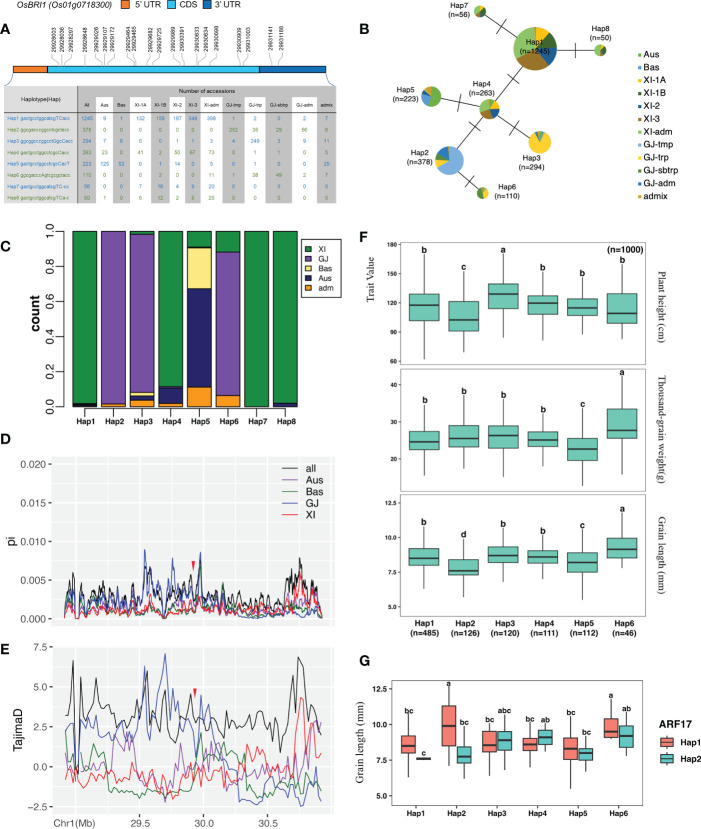
Haplotype analysis of *HFR131* and *OsARF17*. **(A)** Haplotypes of *HFR131*/*OsBRI1*. An uppercase letter in the haplotype sequence indicates a nonsynonymous mutation compared with Hap2. **(B)** Evolutionary network of *HFR131* haplotypes. **(C)** Frequency of *HFR131* haplotypes among subgroups of rice accessions. **(D, E)** Nucleotide diversity (π) and Tajima’s *D* for ∼1 Mb genomic region flanking *HFR131*. The red arrow indicates the position of *HFR131*. **(F)** Distribution of plant height, grain length, and thousand-grain weight among rice accessions with different *HFR131* haplotypes. **(G)** Grain length of rice accessions with different haplotypes of *HFR131* and *OsARF17 Aus*, *Bas*, *XI*, and *GJ* refer to different subgroups of rice and *Adm* (*admix*) is a fully mixed group. *XI-1A*, *XI-1B*, *XI-2*, *XI-3*, and *XI-adm* belong to the *XI* group. *GJ-tmp*, *GJ-trp*, *GJ-sbtrp*, and *GJ-adm* belong to the *GJ* group. Different letters on the boxplots indicate statistically significant differences at *P* < 0.05 based on Duncan’s multiple range test.

To further understand the relationship between *HFR131* and *OsARF17* in regulating plant height and grain length, we downloaded the gcHap data for *HFR131* and *OsARF17* from the RFGB and conducted a haplotype analysis. Eight major Haps (≥30 rice accessions) (Zhang et al., 2021) were resolved based on 20 SNPs distributed among the CDS region and the 3′ untranslated region of *HFR131*, and a haplotype network was constructed (**Figures 6A, B**). *Xian*/*Indica* (*XI*) accessions predominantly harbored Hap1, Hap4, Hap7, and Hap8, whereas *Geng*/*Japonica* (*GJ*) accessions predominantly carried Hap2, Hap3, and Hap6, indicating that there was a strong *XI*–*GJ* differentiation of *HFR131* Haps (**Figures 6B, C**). Nucleotide diversity (π) among different populations revealed that *GJ* harbored higher diversity than *XI*, not only in the gene region of *HFR131*, but also in the ~500 kb upstream/downstream regions of *HFR131* (**Figure 6D**). Furthermore, *HFR131* has not evolved neutrally; the gene was subject to balancing selection in *GJ*, and directional selection or population expansion in *XI* (**Figure 6E**), which indicated that *HFR131* may have contributed to *XI* domestication.

We then investigated the potential associations of major Haps with plant height, grain length, and thousand-grain weight by one-way ANOVA followed by Duncan’s multiple-range tests. Hap3 was associated with the highest average plant height (127.64 ± 18.21 cm), whereas Hap2 was associated with the shortest average plant height (106.18 ± 21.3 cm). Both grain length and thousand-grain weight showed low and high values in Hap5 and Hap6, respectively, inferring that the thousand-grain weight of Hap5 and Hap6 was mainly determined by grain length. Hap7 and Hap8 were not included in the haplotype–trait analysis because the samples with phenotype data for the selected traits were too small (nine and four, respectively) (**Figure 6F**).

As a regulator of *HFR131*, we chose two major haplotypes (Hap1 and Hap2) of *OsARF17* (≥600 rice accessions) to further investigate its influence on *HFR131*. *OsARF17^Hap1^* and *OsARF17^Hap2^* were present in 2680 rice accessions and also showed an unequal distribution between *XI* and *GJ* (**Supplementary Figure 3A**). *OsARF17^Hap1^* was present mostly in *XI*, whereas *OsARF17^Hap2^* was predominantly in *GJ*, especially in *GJ-tmp* (*GJ* from East Asian temperate) and *GJ-trp* (GJ from Southeast Asian tropical) accessions (**Supplementary Figures 3A, B**). We performed two-way ANOVA followed by Duncan’s multiple-range test to determine the consociation between *HFR131* and *OsARF17*, and we observed that a significant consociation only existed when considering grain length. Both *HFR131^Hap2^/OsARF17^Hap1^* and *HFR131^Hap6^/OsARF17^Hap1^* showed an “a” level in multiple comparisons with long grain length (**Figure 6G**). However, the *HFR131^Hap2^/OsARF17^Hap1^* group consisted of only two samples and the grain length of these two samples was divergent (12.7 mm and 7.1 mm, respectively). The grain length of *HFR131^Hap6^* and *OsARF17^Hap1^* was 9.391 mm and 8.567 mm, respectively; however, the grain length of *HFR131^Hap6^/OsARF17^Hap1^* increased to 9.9 mm (**Figure 6G**, **Supplementary Figure 3C**), revealing that the consociation of *OsAFR17^Hap1^*/*HFR131^Hap6^* conferred a longer grain length, and thus improved rice yield.

## Discussion

### The mutation of conserved amino acid in the kinase domain defects the function of *OsBRI1*

In *Arabidopsis thaliana*, BRI1 is a receptor of BR, containing a leucine-rich repeat (LRR) domain, a transmembrane domain, and a kinase domain, of which the kinase domain is crucial for the transduction of BR signals (Li and Chory, 1997). BR binds to the extracellular LRR domain of BRI1, and induces association and transphosphorylation between the kinase domains of BRI1 and the co-receptor kinase BAK1. BRI1 activates BAK1 by phosphorylating the activation loop of BAK1, and BAK1 phosphorylates the juxtamembrane and kinase domains of BRI1 to enhance BRI1 signaling in turn. The BR signal is then transmitted downstream (Wang et al., 2012). It has been reported that OsBRI1 and OsBAK1 have a similar structure and function to BRI1 and BAK1, respectively (Yamamuro et al., 2000; Song et al., 2008; Li et al., 2009). In rice, 11 OsBRI1-associated mutants (*d61-1* to *d61-10* and *Fn189*) have been reported, among which the *Fn189*, *d61-1*, *d61-4*, and *d61-10* mutants harbor mutations in the kinase domain of OsBRI1 (Yamamuro et al., 2000; Nakamura et al., 2006; Zhao et al., 2013). In the present study, we identified a new allele of *OsBRI1*, *hfr131*, in which a new mutation site, T988I, in the kinase domain of OsBRI1 was detected, causing significant reductions in plant height and grain length. We compared the protein sequences of the BR receptor and its homologous genes in *Arabidopsis thaliana*, rice, tomato, pea, and barley, and determined that the mutation site is located in a highly conserved region (**Figure 4C**), indicating that the site is critical for the function of the OsBRI1 kinase domain and therefore for the function of the gene. Serine/threonine and tyrosine residues are the common phosphorylation sites in BRI1 and BAK1 (Wang et al., 2012). However, T988 in the kinase domain of OsBRI1 was changed to I, an amino acid that cannot be phosphorylated, in NIL-*hfr131*, indicating that this change may affect the phosphorylation of OsBAK1 to OsBRI1, thus hindering transduction of the BR signal.

The plant height of rice mainly depends on the elongation of internodes. Dwarf mutants can be categorized into six types according to the elongation pattern of the upper four or five internodes, namely N-type (wild type), dn-type, dm-type, d6-type, nl-type, and sh-type (Yamamuro et al., 2000). Mutants defective in BR synthesis or signaling are often of the dm-type or d6-type. The *d2*, *d11*, and *d61-1* mutants exhibit the dm-type internode elongation pattern (**Supplementary Table 2**), showing a specific shortening of the second internode. The *brd2*, *d61-2*, and *Fn189* mutants show the d6-type internode elongation pattern (**Supplementary Table 2**), in which all the internodes are extremely shortened except for the first internode. However, brd1 failed to elongate any internodes (**Supplementary Table 2**). The T988I conversion in the kinase domain of *OsBRI1*/*HFR131* caused reduction in plant height with equally proportional shortening of all internodes (**Figure 1G**), which represented the dn-type internode elongation pattern and had not been reported previously in BR synthesis- or *OsBRI1*-defective mutants (**Supplementary Table 2**).

### *hfr131* confers the dn-type internode elongation pattern by coordinating different hormones

The formation of different internode elongation patterns may be associated with differences in the main regulatory hormones at different internodes. GA is considered to play an important role in the elongation of the first internode in rice. The first internode of the GA synthesis-defective *d35* mutant is shortened to a significantly greater degree than the other internodes (Itoh et al., 2004). *HOX12* and *EUI* influence elongation of the first internode by regulating the contents of active GAs (Luo et al., 2006; Zhu et al., 2006; Gao et al., 2016). All phenotypic effects of BR result from BR synthesis and perception. BR-synthesis and *OsBRI1*-related mutants will assist in providing an improved understanding of the role of BR in rice internode regulation. All BR-synthesis and *OsBRI1*-related mutants reported to date show severe shortening or non-elongation of the second internode, with dm- or d6-type elongation patterns (**Supplementary Table 2**), indicating that elongation of the second internode may be mainly determined by BR. For the lower internodes, auxin may be the primary functional hormone. Loss-of-function of BR2, which functions in indole-3-acetic acid export (Knöller et al., 2010), disrupts polar auxin transport in the maize stalk, resulting in a reduced plant height with compact lower-stalk internodes (Multani et al., 2003). These findings confirmed that the distribution of auxin plays a crucial role in regulating the lower internodes. In the present study, NIL-*hfr131*, harboring a new allele of *OsBRI1*, *hfr131*, showed a dn-type internode elongation pattern rather than the d6- or dm-type, indicating that this allele can coordinate different hormones. It was further confirmed by transcriptome analysis that *OsGA2ox10*, which participates in the GA catabolic pathways (Lo et al., 2008), was significantly up-regulated and that *OsPIN1d*, *OsPIN8*, and *OsAUX2*, which are auxin transporters (Wang et al., 2009; Yu et al., 2015), were significantly down-regulated (**Supplementary Table 1**), implying that there may be coordination between different hormones.

We noticed that the location of *HFR131*/*OsBRI1* was closed to the famous “Green Revolution” gene *SD1* (Monna et al., 2002; Sasaki et al., 2002; Su et al., 2021), both of which were located on the long arm of chromosome 1, and the distance of them was nearly 8500 kb (**Supplementary Figure 1**). Either *SD1*-mutant or NIL-*hfr131* showed a semi-dwarf plant height, but the expression pattern of them were different. *SD1* was widely expressed in different organs especially in leaf blades (Monna et al., 2002; Sasaki et al., 2002; Su et al., 2021), however, *OsBRI1* was strongly expressed in shoot apex but hardly in leaf blades (Yamamuro et al., 2000), indicating that they regulated plant height through different pathway, also implying that *hfr131* may be another useful dwarfing gene resource after *sd1*.

### *OsARF17*-*HFR131* links brassinosteroid and auxin signaling during regulation of plant height and grain length

Auxin and BR coordinate to regulate many aspects of plant growth and development, such as root and bud development (Mazzoni-Putman et al., 2021), and several studies have shown that there is crosstalk between BR and auxin; for example, exogenous auxin improves the sensitivity of rice to BR (Fujioka et al., 1998), and BR affects the transport of auxin (Symons et al., 2007). With extension of the research scope, ARF family proteins were found to be important factors communicating between the BR and auxin signaling pathways. In *Arabidopsis thaliana*, *ARF2*, *ARF7*, and *ARF19* are involved in BR and auxin communication through different mechanisms (Vert et al., 2008; Sun et al., 2010; Cho et al., 2014). In rice, OsARF11 and OsARF19 can bind to the *OsBRI1* promoter and promote the expression of *OsBRI1*, thus enhancing the rice response to BR and regulating leaf angle (Sakamoto et al., 2013; Zhang et al., 2015). However, how the BR and auxin communication affects plant height and grain size in rice is poorly understood. In the present study, we confirmed that OsARF17 may be involved in the response to BR through *HFR131*. OsARF17 can bind to the promoter region of *HFR131* and positively regulate the expression of *HFR131* (**Figures 5I–K**). *OsARF17* knockout lines showed decreased sensitivity to BR (**Figures 5L–N**). *OsARF17* and *HFR131* defective and overexpression lines showed a similar phenotype. Both *OsARF17* knockout lines and NIL-*hfr131* showed a reduced plant height and grain length, whereas both overexpression lines of *OsARF17* and *HFR131* exhibited an increased grain length, indicating that BR and auxin regulate plant height and grain length synergistically through *OsARF17*–*HFR131* interaction. In addition, *HFR131^Hap6^/OsARF17^Hap1^* in combination conferred a longer grain length than both *HFR131^Hap6^* and *OsARF17^Hap1^* individually. This may be the result of efficient coordination of the BR and auxin signaling pathways between the two haplotypes, and highlights the importance of breaking through the limitation of traditional single-gene breeding to increase rice yield.

In summary, this study identified a new allele of *OsBRI1*, *hfr131*, which caused a significant decrease in plant height and grain length of rice. It was further found that *OsARF17* could regulate the plant height and grain length of rice, and affect the sensitivity of rice to BR, by regulating the expression of *HFR131*. In addition, the *OsAFR17^Hap1^*/*HFR131^Hap6^* consociation was confirmed to contribute to longer grain length. These findings are of great importance for improvement of rice yield by molecular breeding in the future.

## Data availability statement

The data presented in the study are deposited in the GEO repository, accession number GSE224118.

## Author contributions

XL, YW, DL, and LC contributed to conception and design of the study. DL, LC, ML, FM, and PY performed the experiments. DL, LC, XZ, JH, FN, SH, JC, XY, and JY analyzed and interpreted the data. DL, LC, and ML wrote the manuscript and XL and YW revised the manuscript. All authors contributed to the article and approved the submitted version.
