# Supplementary material for: The identification and characterization of a plant height and grain length related gene hfr131 in rice
Source: Front Plant Sci. 2023 Mar 24;14:1152196. doi: 10.3389/fpls.2023.1152196 (PMC10080003; doi:10.3389/fpls.2023.1152196)
Supplement: Supplementary file 1 [file DataSheet_1.docx]

Supplementary Material

The identification and characterization of a plant height and grain length related gene *hfr131* in rice

**Dengyong Lan†, Liming Cao†, Mingyu Liu, Fuying Ma, Peiwen Yan, Xinwei Zhang, Jian Hu, Fuan Niu, Shicong He, Jinhao Cui, Xinyu Yuan, Jinshui Yang, Ying Wang*, Xiaojin Luo***

*** Correspondence:** Xiaojin Luo: [luoxj@fudan.edu.cn](mailto:luoxj@fudan.edu.cn); Ying Wang: [wang_y@fudan.edu.cn](mailto:wang_y@fudan.edu.cn)


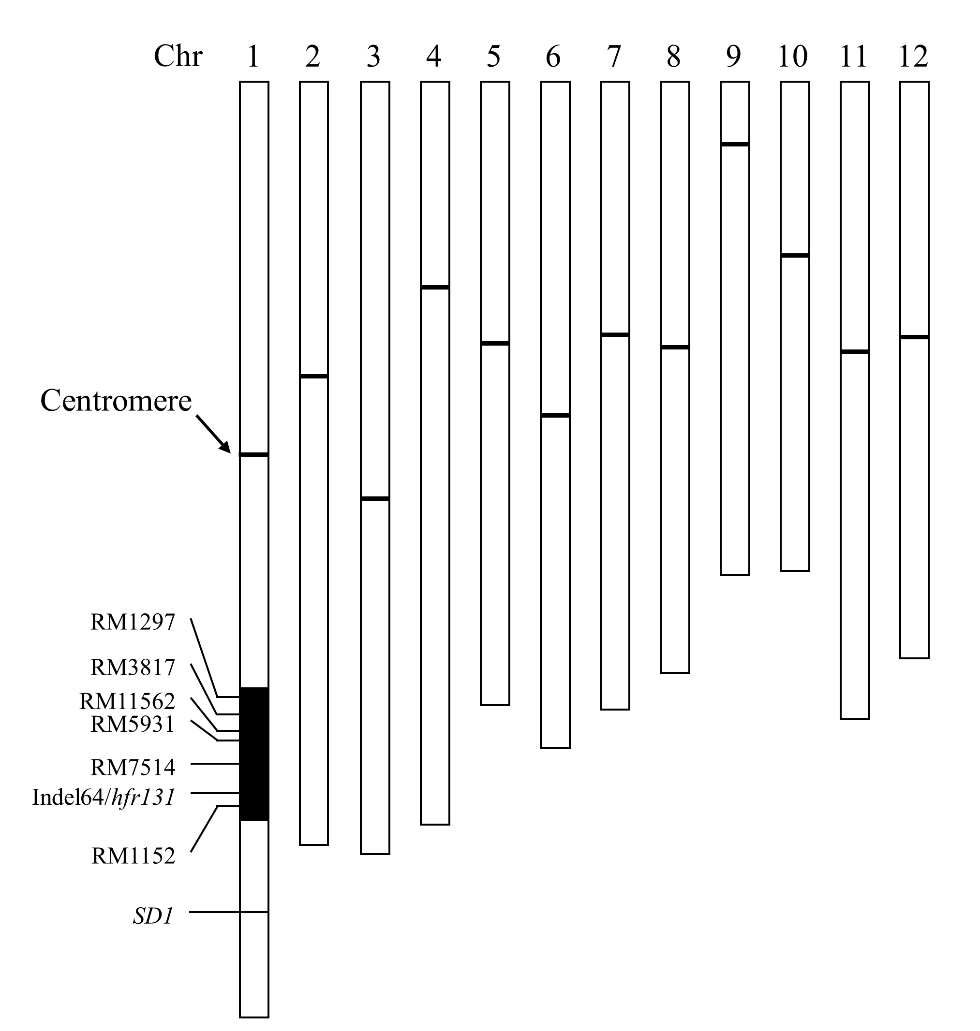


**Supplementary Figure 1.** Schematic diagram of the chromosomes of the near-isogenic line NIL-*hfr131*.


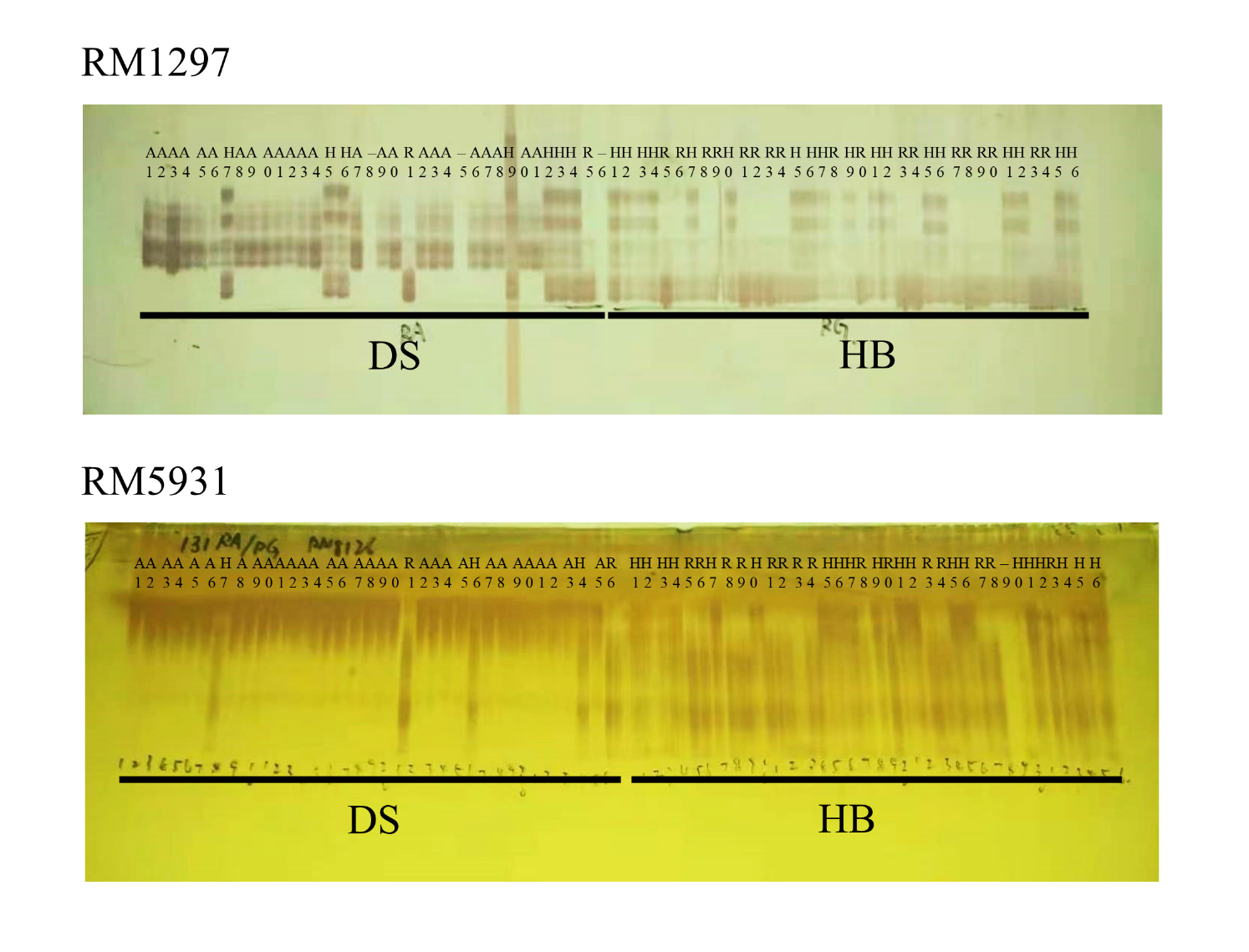


**Supplementary Figure 2.** Electrophoretogram of the markers RM1297 and RM5931. “A” represents the banding pattern of *Oryza longistaminata*, “R” represents the banding pattern of ‘187R’, “H” represents the banding pattern of heterozygote.


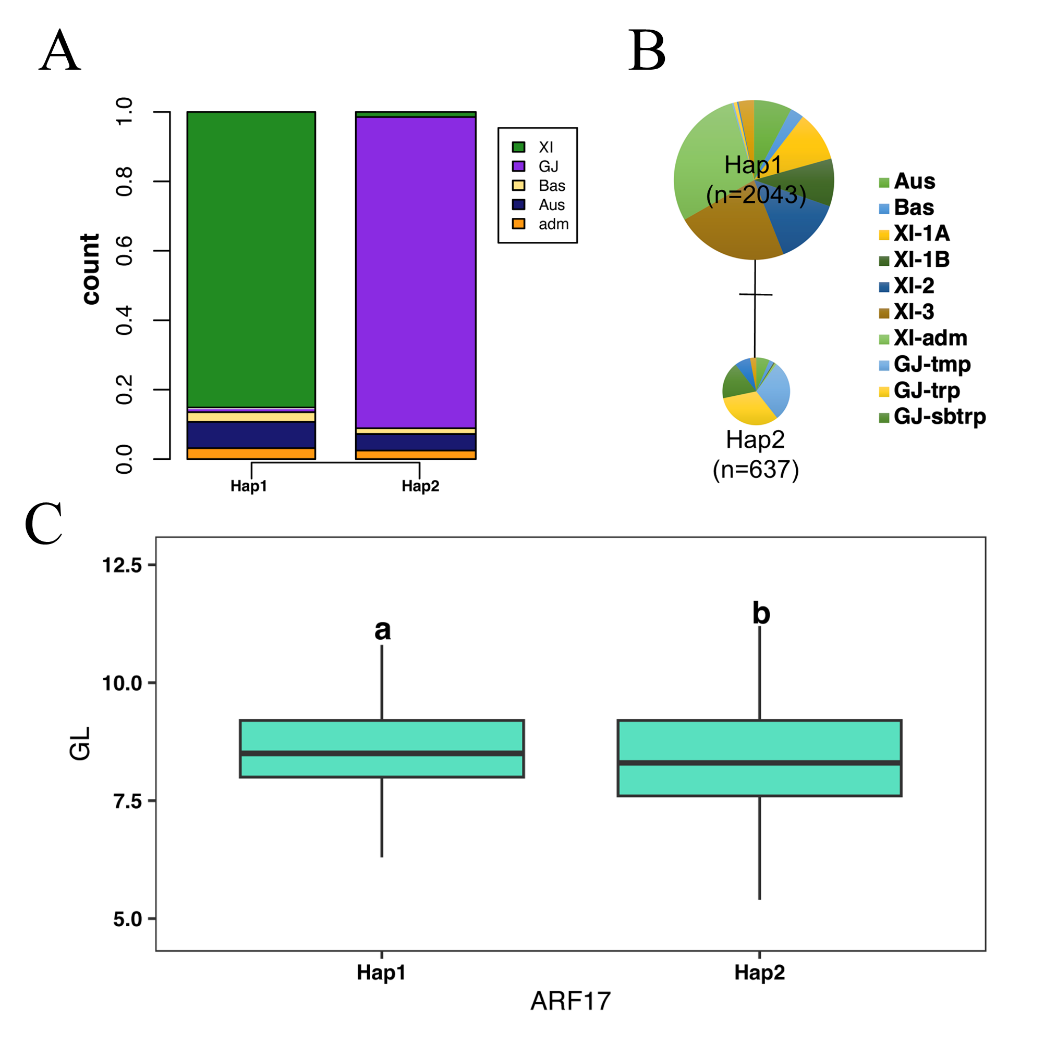


**Supplementary Figure 3.** **(A)** Frequency of different haplotypes of *OsARF17* among the subgroups of rice. **(B)** Evolutionary network of different haplotypes of *OsARF17*. **(C)** Distribution of grain length (GL) among different haplotypes of *OsARF17*. Different letters on the boxplots indicate statistically significant differences at *P* < 0.05 based on Duncan’s multiple range test.

**Supplementary Table 1. Genes associated with *HFR131* function identified in the transcriptome data***

| Name | Gene id | MeanTPM (B) | MeanTPM (A) | log2FoldChange | pValue | qValue | result |
| --- | --- | --- | --- | --- | --- | --- | --- |
| *D2* | LOC_Os01g10040 | 19.11489 | 13.68309 | 0.482302 | 0.01501 | 0.342426 | up |
| *D11* | LOC_Os04g39430 | 44.10432 | 20.96671 | 1.072819 | 3.32E-12 | 1.94E-09 | up |
| *BRD1* | LOC_Os03g40540 | 43.94496 | 28.14821 | 0.642655 | 0.002685 | 0.122263 | up |
| *GS2* | LOC_Os02g47280 | 14.62136 | 20.49443 | -0.487155 | 0.024509 | 0.417041 | down |
| *OsLG3* | LOC_Os03g08470 | 17.91541 | 25.51796 | -0.510312 | 0.000509 | 0.03975 | down |
| *OML4* | LOC_Os02g31290 | 131.3525 | 73.46369 | 0.83834 | 0.047075 | 0.526662 | up |
| *OsREM4.1* | LOC_Os07g38170 | 47.95114 | 33.90867 | 0.499911 | 0.014606 | 0.339235 | up |
| *OsAP2-39* | LOC_Os04g52090 | 162.9664 | 101.116 | 0.688563 | 2.20E-06 | 0.000455 | up |
| *OsCYP96B4* | LOC_Os03g04680 | 10.03463967 | 6.635959333 | 0.596611862 | 0.007519023 | 0.237643973 | up |
| *LHD2* | LOC_Os01g68000 | 0.0001 | 0.069779 | -9.446649 | 0.021396 | 1 | down |
| *OsGA2ox10* | LOC_Os05g11810 | 1.716454 | 0.987436 | 0.797671 | 0.008957 | 0.265521 | up |
| *OsPIN1d* | LOC_Os12g04000 | 2.071688 | 3.022951 | -0.545151 | 0.019344 | 0.379614 | down |
| *OsPIN8* | LOC_Os01g51780 | 4.519382 | 6.60273 | -0.546937 | 0.014745 | 0.339386 | down |
| *OsAUX2* | LOC_Os03g14080 | 9.359471 | 13.99098 | -0.579998 | 0.003421 | 0.145271 | down |

* The transcriptome datasets were available from the GEO database as accession nos. GSE224118.

**Supplementary Table 2. Internode elongation pattern of** **brassinosteroid synthesis- and *OsBRI1*-related mutants**

| Mutants | | Pattern* | | References |
| --- | --- | --- | --- | --- |
| BR synthesis | *d2* | dm-type | (Hong et al., 2003) | |
|  | *d11* | dm-type | (Tanabe et al., 2005) | |
|  | *brd1* | None of the internodes were elongated | (Hong et al., 2002) | |
|  | *brd2* | d6-type | (Hong et al., 2005) | |
| *OsBRI1* | *d61-1* | dm-type | (Yamamuro et al., 2000) | |
|  | *d61-2* | d6-type | (Yamamuro et al., 2000) | |
|  | *Fn189* | d6-type | (Zhao et al., 2013) | |
|  | *hfr131* | dn-type | In this study | |

* The pattern is based on the information and description of internodes in the corresponding reference

**Supplementary Table 3. Primers used in this study**

| Name | Forward（5’-3’） | Reverse（5’-3’） | Product size | Purpose |
| --- | --- | --- | --- | --- |
| RM1297 | GTGCCTTACAACTCAACGAC | CACTCCCAGTTCAGTACGTC | 169 bp | For fine mapping |
| RM3817 | AAGGAAGGGGAAGCTGAGAG | CGGACTCGAGAAAAAAGCAG | 207 bp |  |
| RM11562 | TGCACTGACCTAGTTCGATATTGC | CATTCGAGAGATGCAACAACACC | 183 bp |  |
| RM5931 | CTCTCCTGCCTGACAAAAGC | GTTTTAGCGGATGTGGCATC | 193 bp |  |
| RM1152 | GCCTTTGTCCTTCAGTAGGC | AGAGCGCCTGGGTATAATTG | 183 bp |  |
| RM7514 | AACACTCGCGAGTCGCAAC | GTGTATAGGGGGATGGGGAG | 173 bp |  |
| Indel 64 | ACAAGGAATCCATGTACGAGC | GGATTTAACCTCGCCGTAAGC | 798 bp/365 bp |  |
| ProHFR131 | CTGAACTCCTCGAGCAGCTG | CATGAATTAGCAGCTGCGTACG | 3705 bp/3067 bp | For Dual-luciferase reporter assay |
| pGreen-HFR131 | GGCCCCCCCTCGAGGTCGACGGAGGAATTTTGAATGGC | GCTCTAGAACTAGTGGATCCGTACGAGCGAGCTCACTG | 3540 bp/3107 bp |  |
| ARF17KO | CTTGGTCTTCTGACCAACTGC | ATGCGTACTTGTGTCACTTGC | 739 bp/733 bp/697 bp | For detecting the knock-out mutants of *OsARF17* |
| ARF17OE | AGCTCGGTACCCGGGGATCCATGAGGCTTTCGTCGTCG | CCAAGGGCGAATTGGTCGACGAATTCAACTGAGCCGAC | 2794 bp | For constructing overexpression lines |
| BRI1OE | AGCTCGGTACCCGGGGATCCATGGATTCCTTGTGGGCA | CCAAGGGCGAATTGGTCGACATCCTTCTCCTCCTTGGC | 3406 bp |  |
| Actin | CATTGGTGCTGAGCGTTTCC | TCCTTGCTCATCCTGTCAGC | 201 bp | For qPCR and Chip-qPCR |
| UBQ5 | ACCACTTCGACCGCCACTACT | ACGCCTAAGCCTGCTGGTT | 69 bp | For qPCR |
| qHFR131 | ATAATCAGCTGGACTCGGCG | TCTCCGACAAGGAAAGTGCC | 201 bp |  |
| qARF17 | ATGAGGCTTTCGTCGTCGTC | AGCTACCTGTTCGCTGTGAC | 183 bp |  |
| pAbAi-A1 | TTGAATTCGAGCTCGGTACCTGCGTCTTTCTTCTTCGC | GCACATGCCTCGAGGTCGACGAGCTCACTGCCTCACGA | 190 bp | For yeast one-hybrid assay |
| pAbAi-A2 | TTGAATTCGAGCTCGGTACCAGTCCGGTCAATCCGGCG | GCACATGCCTCGAGGTCGACTTAAGTATTCTAATCCCC | 190 bp |  |
| pAbAi-A3 | TTGAATTCGAGCTCGGTACCGAGCCAAGCCGAGACTAG | GCACATGCCTCGAGGTCGACCGGTGGAGGCTGCGGCGT | 190 bp |  |
| pAbAi-A4 | TTGAATTCGAGCTCGGTACCCGGCTTAAATACCAAACG | GCACATGCCTCGAGGTCGACCTTCACTAGAAAATAAAA | 190 bp |  |
| pAbAi-A5 | TTGAATTCGAGCTCGGTACCCTGTCAGGGCAGGGAAAC | GCACATGCCTCGAGGTCGACGAGCCAAGCTAAGCGAGC | 190 bp |  |
| pAbAi-A6 | TTGAATTCGAGCTCGGTACCTTAGGTTGATTTTGTCAG | GCACATGCCTCGAGGTCGACCCTACCCGCAAAAGAAAG | 340 bp |  |
| pAbAi-A7 | TTGAATTCGAGCTCGGTACCAGAGAACTTTTACTTAAT | GCACATGCCTCGAGGTCGACTAGTATAATATCTATAGT | 190 bp |  |
| pAbAi-A8 | TTGAATTCGAGCTCGGTACCTTACATAAAACACTTAAT | GCACATGCCTCGAGGTCGACCTATGTATTAAGTAAAAG | 190 bp |  |
| Y1-test | AGAACGAAGGAAGGAGCACAG | GGTTCTGGCGAGGTATTGGAT | 1100 bp |  |
| pAbAi | CCTTCTGTTCGGAGATTACCG | AGATGTAATGCACCCAGTCGC | Depend on the fragment inserted |  |
| AD-ARF17 | ACGTACCAGATTACGCTCATATGAGGCTTTCGTCGTCG | AGCTCGAGCTCGATGGATCCTCAGAATTCAACTGAGCC | 2794 bp |  |
| ChIP-A5 | GATCAGGCGATCACTGCACC | CTAAGCGAGCTCGAGCCAAG | 99 bp | For ChIP-qPCR |
| ChIP-A6 | CTGTCTCAGGTCATACCAAGGAAG | CAGGCATGAGATCGTCACACG | 215 bp |  |
| ChIP-A7 | CGGTTGCCGGTAAGCAATACC | GCAGTCCAATGCTGCACTGG | 229 bp |  |
